# Effects of Rational-Emotive Hospice Care Therapy on Problematic Assumptions, Death Anxiety, and Psychological Distress in a Sample of Cancer Patients and Their Family Caregivers in Nigeria

**DOI:** 10.3390/ijerph13090929

**Published:** 2016-09-20

**Authors:** Kay Chinonyelum Nwamaka Onyechi, Liziana N. Onuigbo, Chiedu Eseadi, Amaka B. Ikechukwu-Ilomuanya, Okechukwu Onyinye Nwaubani, Prince C.I. Umoke, Fedinand U. Agu, Mkpoikanke Sunday Otu, Anthonia N. Utoh-Ofong

**Affiliations:** 1 Department of Educational Foundations, Faculty of Education, University of Nigeria, Room 213, Harden Building, Nsukka 410001, Nigeria; kay.onyechi@unn.edu.ng (K.C.N.O.); liziana.onuigbo@unn.edu.ng (L.N.O.); amaka.ikechukwu-ilomuanya@unn.edu.ng (A.B.I.-I.); mkpoikankeotu@gmail.com (M.S.O.); 2 Department of Social Science Education, University of Nigeria, Nsukka 410001, Nigeria; okechukwu.nwaubani@unn.edu.ng; 3 Department of Human Kinetics and Health Education, Faculty of Education, University of Nigeria, Nsukka 410001, Nigeria; prince.umoke@unn.edu.ng (P.C.I.U.); fedinand.agu@unn.edu.ng (F.U.A.); 4 Quality Assurance Department, National Examination Council, Minna 920211, Nigeria; toniautoh@yahoo.com

**Keywords:** cancer patients, death anxiety, family caregivers, psychological distress, rational emotive hospice care therapy

## Abstract

This study was a preliminary investigation that aimed to examine the effects of rational emotive hospice care therapy (REHCT) on problematic assumptions, death anxiety, and psychological distress in a sample of cancer patients and their family caregivers in Nigeria. The study adopted a pre-posttest randomized control group design. Participants were community-dwelling cancer patients (*n* = 32) and their family caregivers (*n* = 52). The treatment process consisted of 10 weeks of full intervention and 4 weeks of follow-up meetings that marked the end of intervention. The study used repeated-measures analysis of variance for data analysis. The findings revealed significant effects of a REHCT intervention program on problematic assumptions, death anxiety, and psychological distress reduction among the cancer patients and their family caregivers at the end of the intervention. The improvements were also maintained at follow-up meetings in the treatment group compared with the control group who received the usual care and conventional counseling. The researchers have been able to show that REHCT intervention is more effective than a control therapy for cancer patients’ care, education, and counseling in the Nigerian context.

## 1. Introduction

Cancer is one of the most common terminal and life-threatening illness and comes with huge psychosocial and economic burdens [1,2,3]. Its diagnosis presents a major episode for the person diagnosed and also for the family caregivers and other family members [4]. Though previous studies indicate that a cancer diagnosis actually has a greater impact on the family members than on the patient [5,6,7,8,9,10,11], cancer patients generally have additional physical, social, spiritual, and emotional requirements, and they may experience pronounced physical symptoms, social isolation, and feelings of spiritual abandonment, sadness, anxiety, helplessness, anger, and guilt [12]. The emotional and economic impacts of a cancer diagnosis are intricate enough to handle without the added burden of having to travel long distances away from home for cancer care, with or without family, friends, neighbors, or other social support networks [13,14]. However, given outpatient care, longer survival, and patients’ wishes to be cared for at home, most cancer care is usually community based [15].

Just as in other parts of the world, cancer is a major public health issue in Nigeria [16,17]. A study by Jedy-Egba et al. [18] showed that the incidence of cancer in Nigeria was on the rise. According to Doka [19], a life-threatening illness (such as cancer) is a multifaceted crisis that affects an individual physically, psychologically, socially, financially, and spiritually. Thus, effective community-based and/or home-based counseling and caregiving might be required to respond to these dimensions of crisis. If so, then a hospice care intervention based on the rational emotive behavior therapy (REBT) approach is warranted.

The REBT theory developed by Albert Ellis in 1955 is used by therapists to deal with problems of human disturbance and is based on the concept that people primarily disturb themselves, which suggests that they—with the help of a therapist—can take action to reduce that disturbance [20,21,22,23]. According to Ellis and Bernard [23], rational emotive behavior therapists are able to show to people how their attitudes and beliefs are responsible for their emotional distress and interpersonal problems, and this awakens in them the hope that they have some control over their destiny. It is possible that REBT philosophies and methodologies can be extended to provide hospice care for cancer patients in the advanced stage of their illness and to their family caregivers.

In this study, the rational emotive hospice care therapy (REHCT), an adaptation of the REBT theory, is a home-based palliative care program and/or hospice care counseling intervention aimed at alleviating the problematic assumptions, death anxiety, and psychological distress of cancer patients and their family caregivers. According to Sobel [24], all the principles of REBT theory can be appropriately and humanistically applied to a dying patient in that the same problematic assumptions that lead healthy patients into emotional difficulty appear in the thinking of people coping with life-limiting and life-threatening illnesses. REHCT is, therefore, informed by REBT theory in that it is meant to help dying patients and their caregivers in coping and avoiding self-defeating thoughts. Furthermore, the argument for an REHCT approach is based on the notion that grief itself can be extremely debilitating and creates a downward emotional spiral, impacting relationships and work [25]. In fact, relentless grief can result in a host of physical, emotional, and psychological problems such as depression, death anxiety, health anxiety, and panic attacks [25]. It should be noted, however, that the effectiveness of the therapeutic encounter is enhanced when clients understand the rationale of the treatment and are committed to carrying out the tasks specified in their treatment plan [25,26].

There is evidence in the psychological literature that REBT theory, with its cognitive, emotive, and behavioral techniques, can be effectively used to help most clients regain a sense of control, while helping others to adjust to life after the death of a spouse, friend, or family member [25,27,28,29,30]. According to McLeod [31], the behavior element of the therapy may involve setting homework for the client to do—asking the caregivers to keep a diary of thoughts, for example. Without giving false hope or optimism, the REHCT therapist also mobilizes the patient’s will to live, encourages the expression and growth of the self, and facilitates the patient’s self-actualization [32,33,34].

From the rational thanatologist’s perspective, a well-designed REHCT intervention can bring about an adaptive and creative control that reinforces life options in the face of death, in direct relation to a dying patient’s willingness to make decisions and dispute problematic assumptions [24]. The REHCT therapist acknowledges that a step-by-step decision-making process can be applied to one particular problem at a time during the therapeutic encounter [35] and that, within a short time, the therapy can achieve the following goals with regard to end-of-life decision-making: the examination of a patient’s deficits in decision-making, self-observation, and reinforcement of a “vigilant” style of problem-solving [36].

As a family system approach, REHCT conceives the entire family, not just the terminally ill patient, as the recipient of therapy. Therefore, during REHCT, family members are encouraged to understand and express their feelings in anticipation of the death of their loved one [37]. Researchers feel that REHCT offers an experience that may enable everyone in the family to accept the facts and work together to enhance the quality of life of the dying cancer patient.

However, despite the available rational emotive and cognitive behavioral resources and techniques that Nigerian therapists could use to assist the terminally ill patients and their bereaved family members and caregivers, it is still unknown whether an REHCT approach could be helpful in assisting individuals in Nigeria. This study aims to answer that question by examining the effects of REHCT on the problematic assumptions, death anxiety, and psychological distress in a sample of cancer patients and their family caregivers in Nigeria.

## 2. Materials and Methods

### 2.1. Ethical Approval

The ethical approval for conducting this study was obtained from the Faculty of Education Research Grants Committee, University of Nigeria, Nsukka. (Ethical approval code: UNN/FE/R.2).

### 2.2. Participants

The participants were terminal cancer patients and their family caregivers from 80 households in the south-eastern part of Nigeria. The researchers had an accessible household population of 80 cancer patients with 365 family members. From these, 32 households qualified for the study—32 cancer patients and 52 family members met the inclusion criteria and took part in the study. The sociodemographic information of the participants is shown in Table 1.

### 2.3. Procedure

The intervention program was advertised purposively at various medical centers, educational settings, religious services, and social gatherings in the south-eastern part of Nigeria for up to 7 months between November 2014 and May 2015. In this time, the researchers received invitations from 80 households to carry out the intervention program with them. The researchers performed screening for the participants by putting certain limitations on recruitment. The study’s inclusion criteria included: being at the terminal stage of breast, cervical, or prostate cancers; having finished cancer treatment and not receiving other therapeutic treatment; having a family caregiver who is a very close relative; with family caregivers available throughout the program. The participants also had to have scores within set benchmark values for problematic assumptions (56–75, a high level of problematic assumption), death anxiety (56–75, a high level of death anxiety), and psychological distress (30–50, a severe level of psychological distress). These were ascertained from self-report questionnaires and/or structured interviews.

**Figure 1:**
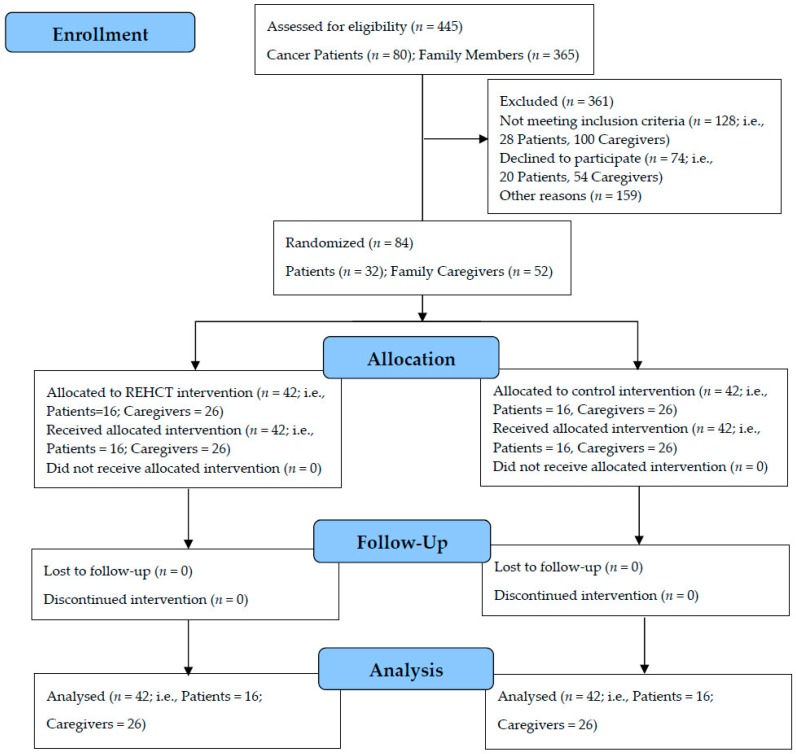
Flow diagram of the participant eligibility criteria.

The community-dwelling terminal cancer patients (*n* = 32) and some of their family caregivers (52) were identified as having problematic assumptions, death anxiety, and psychological distress, and met the inclusion criteria. Therefore, 84 participants were selected to take part in the study. The participants were assigned to control and treatment groups by simple randomization. (see Figure 1).

To carry out the randomization, the researchers used envelopes which were numbered in advance and opened sequentially, only after a participant’s name was written on the appropriate envelope. The envelopes contained pressure sensitive paper that enabled the transfer of information to the assigned allocation.

The participants’ written informed consent was obtained. The researchers also developed a manual (the REBT Hospice Care Manual) to guide the treatment process, which consisted of 10 weeks of full intervention and 4 weeks of follow-up meetings that marked the end of the program. The REHCT sessions lasted for up to 45 min and were held once a week for each household. Overall, the participants in the treatment group received 10 sessions of REHCT each.

All the participants completed self-report questionnaires and/or structured interview schedules at three time points (Time 1, Time 2, and Time 3). At Time 1, participants in both groups were pretested before the administration of the intervention package (REHCT) to the treatment group. At Time 2, approximately 10 weeks later, all participants at their different households were assessed by the researchers using self-report questionnaires and/or the structured interviews. The researchers then conducted follow-up meetings over a 4-week period with all the participants at their different homes, leading to a third assessment (Time 3), which also involved completing the self-report questionnaires and/or the structured interviews.

It is worth noting that the four follow-up sessions provided the patients and their family caregivers the opportunity to share with the researchers how they were able to put into effective use the adaptive and rational coping skills of the REHCT. The follow-up sessions enabled the researchers to assess the participants’ progress in treatment and to monitor efficacy and possible relapse. The follow-up sessions were also an opportunity for the researchers to re-educate the patients and caregivers, check that they remembered the key information gained during the intervention, and ensure that they were adhering to the correct behavior change schedule.

Participants in the control group received the usual care and conventional counseling throughout the intervention period, involving 10 weekly sessions of up to 45 min. The “usual care” consisted of providing daily spiritual support, caregiving, and counseling to the control group participants, along with comforting, advising, encouraging, and reassuring the patients and family caregivers, as well as listening attentively and sympathetically. There were four weekly follow-up sessions for each household.

The researchers employed the services of six research assistants with backgrounds in oncology nursing during the course of this study (two assistants per state) for the purpose of data collection. The researchers and the assistants went to the participants’ respective homes/households to deliver the intervention program throughout the period of the study. The participants did not meet together as a group, only in individual households. Thus, the intervention was not an individualized approach but a family-centered approach to REHCT. The REHCT sessions were delivered in the participants’ native language (Igbo). Igbo is one of the three major Nigerian languages spoken by the majority of people in the south-eastern part of the country.

Given their formal training as guidance counselors with expertise in the principles and practice of REBT theory, four of the authors delivered the treatment intervention whereas the control intervention was delivered by 16 community-based counselors with formal training in guidance and counseling. The participants in both treatment and control conditions returned the questionnaires directly to the researchers after each assessment.

### 2.4. Measures

The self-administered and interviewer-administrated Igbo versions of all the instruments were used in this study. The self-administered versions were given to the family caregivers while the interviewer-administrated versions were used for eliciting responses from the cancer patients with the help of the research assistants. The three sets of questionnaires were validated in Igbo.

**Cancer Patients’ and Family Caregivers’ Assumptions Questionnaire (CPFCAQ):** The CPFCAQ is an Igbo version questionnaire with 15 items that helps to assess the cancer patients’ and family caregivers’ level of problematic assumptions. The CPFCAQ was developed by the researchers for the purpose of this study. The CPFCAQ is structured using a five-point rating scale ranging from “I disagree strongly” to “I agree strongly”. An example of an item is: “It would be awful if I died this way.” Scores range between 15 and 75, where low problematic assumptions = 15–35, moderate problematic assumptions = 36–55 and high problematic assumptions = 56–75. All the items were subjected to confirmatory factor analysis. The self-administered and interviewer-administrated versions of the instrument were used in this study. After pilot testing among the current study participants, the CPFCAQ (self-administered) and the CPFCAQ (interviewer-administrated) versions showed a good internal consistency of 0.87 and 0.89 α respectively for a sample of 40 family caregivers and 30 cancer patients.

**Death Anxiety Questionnaire (DAQ):** This is an Igbo version of a 15-item questionnaire adapted from the death anxiety questionnaire by Conte et al. [38] to measure the extent of death anxiety that a cancer patient and their caregivers experience. The original version of the DAQ is only self-administered and is constructed on a three-point rating of 0 (not at all), 1 (somewhat) and 3 (very much). However, the Likert-type version of the DAQ used in this study provides a rating on a scale ranging from 1 (none of the time) to 5 (all of the time). The score ranges between 15 and 75, where low death anxiety = 15–35, moderate death anxiety = 36–55, and high death anxiety = 56–75. The self-administered and interviewer-administrated versions of the instrument were used in this study. After pilot testing among the current study participants, the test–retest reliability of the DAQ in this study was 0.84 while internal consistency was 0.85 α using 30 cancer participants. The DAQ showed a test–retest reliability of 0.80 and internal consistency of 0.86 α using 40 family caregivers.

**Kessler Psychological Distress Scale (*K*_10_):** The *K*_10_ is a well-validated 10-item instrument designed by Kessler et al. [39] to measure psychological distress (depression and anxiety). The self-administered and interviewer-administrated Igbo versions of the instrument were used in this study. The *K*_10_ is a brief, simple, and reliable instrument for detecting the mental health conditions of patients. Each question in the *K*_10_ pertains to an emotional state and each has a five-level response scale ranging from “none of the time” to “all of the time”. In scoring the *K*_10_, the researchers considered 1 as the minimum score for each item (none of the time) and 5 as the maximum score (all of the time). The sum of these scores yields a minimum possible score of 10 (all answers were “none of the time”) and a maximum possible score of 50 (all answers were “all of the time”). As grouped and categorized in this study, a score of 10–19 indicates that the participant may currently not be experiencing significant feelings of distress; a score of 20–24 indicates that the participant may be experiencing mild levels of distress consistent with a diagnosis of a mild depression and/or anxiety disorder; a score of 25–29 indicates that the participant may be experiencing moderate levels of distress consistent with a diagnosis of a moderate depression and/or anxiety disorder; while a score of 30–50 indicates that the patient may be experiencing severe levels of distress consistent with a diagnosis of a severe depression and/or anxiety disorder. After pilot testing among the current study participants, the *K*_10_ used in this study had an internal reliability consistency of 0.93 α conducted using 30 cancer participants. The *K*_10_ version for caregivers had an internal reliability consistency of 0.90 α and was conducted using 40 family caregivers.

### 2.5. Intervention Package

**REBT Hospice Care Manual (REBT-HCM):** The REHCT manual was made by incorporating the techniques and descriptions in other REBT intervention manuals, which were successfully tested in randomized control trials [40,41,42]. The manual is based on a cognitive behavioral approach, incorporating some of Albert Ellis’s quotes to aid in disputing participants’ problematic assumptions and to motivate participants to change their dysfunctional emotions and thoughts around cancer. Being aware that most cancer patients, especially highly distressed ones, might refuse to make realistic decisions that would combat destructive emotions, the researchers included Sobel’s seven-step decision-making process for the participants in the REHCT intervention program manual [35]. The step-by-step decision process is applied to one specific concern at a time and, as the patient confronts and resolves successive concerns, denial decreases and the desire to live, despite approaching death, follows. The steps include: identification of primary affects; definition of the uppermost problems and subsidiary concerns; generating alternatives and observing covert structures; imagination of how others might respond if asked to deal with similar problems; consideration of the pros and cons of each proposed solution; ranking in order all possible solutions; selection of the most feasible solution; and re-examination and redefinition of the main problem in the light of the assessment.

The manual indicates treatment sessions anchored on several treatment strategies, including cognitive restructuring, confrontation, therapeutic alliance, acceptance, Socratic dialogue, reframing, metaphors, worksheets, and motivation, which form the standard of care for the patients and caregivers in the treatment group. Thus, the REHCT manual borrows evidence-based techniques from other therapeutic approaches such as relaxation techniques, multigenerational family therapy, solution-focused brief therapy, imagery work, and Gestalt therapy. With the exception of the first session, all other sessions usually begin with the question: “What has really changed in the participants?” With this question, the therapeutic tone is set for an expectation of change and a structure to the sessions is provided.

Through expert-consensus procedure, three REBT experts checked the validity of the contents of this manual, which was written in the Igbo language.

### 2.6. Design

The study adopted a pre-posttest randomized control group design.

### 2.7. Data Analysis

The researchers used repeated-measures analysis of variance (ANOVA) to measure at Time 1 (before intervention), at Time 2 (end of the intervention), and at Time 3 (follow-up meetings) the participant’s level of problematic assumptions, death anxiety, and psychological distress, as well as improvements over time across the treatment and control group participants. In other words, a repeated-measures ANOVA (three repeated assessments, time as a within-subjects factor, group as a between-subjects factor) was conducted on outcome variables. The researchers further reported partial eta squared ($\eta_{p}^{2}$) for this design [43]. Prior to analysis, the researchers ensured that there were no missing values and screened for assumption violations with the SPSS 16 software (SPSS Inc., Chicago, IL, USA).

## 3. Results

Table 1 show that the mean age of the cancer patients in the treatment group was 48.33 ± 6.17 years while that of the control group was 48.32 ± 6.43 years. Out of the 32 cancer patients who took part in the study, those in the treatment group comprised of 2 (12.50%) male participants and 14 (87.50%) female participants; while the control group comprised of 2 (12.50%) male participants and 14 (87.50%) female participants. Out of those in the treatment group, 10 (62.50%) had breast cancer, 3 (18.75%) had cervical cancer, and 3 (18.75%) had prostate cancer. For the cancer patients in the control group, 10 (62.50%) had breast cancer, 5 (31.25%) had cervical cancer, and 1 (6.25%) had prostate cancer. Furthermore, the data in Table 1 show that the mean age of the primary caregivers in the treatment group was 55.75 ± 3.03 years while that of the control group was 55.74 ± 3.05 years. Out of the 52 family caregivers who took part in the study, those in the treatment group comprised of 4 (15.38%) male participants and 22 (84.62%) female participants; while the control group comprised of 4 (15.38%) male participants and 22 (84.62%) female participants (see Table 1).

The researchers sought to find out the level of problematic assumptions, death anxiety, and psychological distress in cancer patients and their family caregivers assigned to the control and treatment groups at baseline.

Firstly, the overall magnitude of CPFCAQ scores indicated that problematic assumptions were high in the sample. The tests indicated that there were no baseline differences on problematic assumptions between patients in the treatment and control conditions: *F* (1, 30) = 0.17, *p* = 0.68, $\eta_{p}^{2}$ = 0.01 (see Table 2). Likewise, no baseline differences were found for family caregivers: *F* (1, 50) = 0.03, *p* = 0.86, $\eta_{p}^{2}$ = 0.00 (see Table 3).

Secondly, the overall magnitude of DAQ scores indicated that death anxiety was high in the sample. The tests indicated that there were no baseline differences in death anxiety between patients in treatment and control conditions: *F* (1, 30) = 0.01, *p* = 0.91, $\eta_{p}^{2}$ = 0.00 (see Table 2). Likewise, no baseline differences were found for family members: *F* (1, 50) = 2.93, *p* = 0.13, $\eta_{p}^{2}$ = 0.05 (see Table 3).

Thirdly, the overall magnitude of *K*_10_ scores indicated that psychological distress was severe in the sample. The tests indicated that there were no baseline differences in psychological distress between patients in treatment and control conditions: *F* (1, 30) = 1.90, *p* = 0.18, $\eta_{p}^{2}$ = 0.06 (see Table 2). Likewise, no baseline differences were found for family members: *F* (1, 50) = 0.21, *p* = 0.65, $\eta_{p}^{2}$ = 0.00 (see Table 3).

The researchers conducted repeated-measures ANOVA to find out the effects that a REHCT intervention program would have on problematic assumptions, death anxiety, and psychological distress among the cancer patients and their family caregivers both at the end of the intervention and in follow-up meetings, comparing the treatment group with the control group.

The results show that the REHCT intervention program had a significant effect on problematic assumptions among the cancer patients in the treatment group at the end of the intervention: *F* (1, 30) = 1081.90, *p* = 0.00, $\eta_{p}^{2}$ = 0.98, and remained consistent at follow-up meetings: *F* (1, 30) = 15,919.26, *p* = 0.00, $\eta_{p}^{2}$ = 0.99, compared with those in the control group (see Table 2). Furthermore, the REHCT intervention program had a significant effect on problematic assumptions among the family caregivers in the treatment group at the end of the intervention: *F* (1, 50) = 831.45, *p* = 0.00, $\eta_{p}^{2}$ = 0.95, and was maintained at follow-up meetings: *F* (1, 50) = 28,962.88, *p* = 0.00, $\eta_{p}^{2}$ = 0.99, compared with those in the control group (see Table 3).

Secondly, the results also show that the REHCT intervention program had a significant effect on death anxiety among the cancer patients in the treatment group at the end of the intervention: *F* (1, 30) = 851.80, *p* = 0.00 $\eta_{p}^{2}$ = 0.97, and was maintained at follow-up meetings: *F* (1, 30) = 11,213.52, *p* = 0.00, $\eta_{p}^{2}$ = 0.99, compared with those in the control group (see Table 2). The REHCT intervention program had a significant effect on death anxiety among the family caregivers in the treatment group at the end of the intervention: *F* (1, 50) = 608.83, *p* = 0.00, $\eta_{p}^{2}$ = 0.93, and remained consistent at follow-up meetings: *F* (1, 50) = 16,194.57, *p* = 0.00, $\eta_{p}^{2}$ = 0.99, compared with those in the control group (see Table 3).

Thirdly, the results show that the REHCT intervention program had a significant effect on psychological distress among the cancer patients in the treatment group at the end of the intervention: *F* (1, 30) = 1094.14, *p* = 0.00, $\eta_{p}^{2}$ = 0.98, and at follow-up meetings: *F* (1, 30) = 2676.16, *p* = 0.00, $\eta_{p}^{2}$ = 0.99, compared with those in the control group (see Table 2). The intervention program also had a significant effect on psychological distress among the family caregivers in the treatment group at the end of the intervention: *F* (1, 50) = 963.96, *p* = 0.00, $\eta_{p}^{2}$ = 0.95, and remained consistent at follow-up meetings: *F* (1, 50) = 3741.25, *p* = 0.00, $\eta_{p}^{2}$ = 0.99, compared with those in the control group (see Table 3).

**Figure 2:**
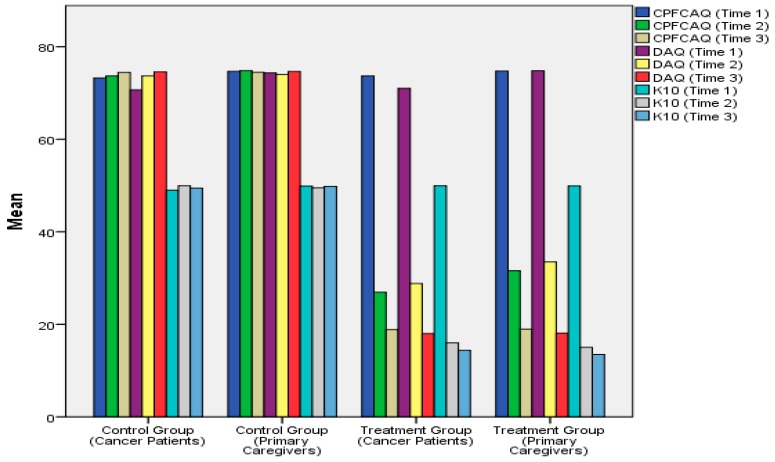
Bar chart showing the effects of rational emotive hospice care therapy (REHCT) intervention on problematic assumptions, death anxiety, and psychological distress in the cancer patients and their family caregivers over time.

Figure 2 shows how the level of baseline problematic assumptions, death anxiety, and psychological distress in cancer patients and their family caregivers that were randomly assigned to the treatment group significantly declined over time due to participation in the REHCT intervention program. The significant improvements were observed at two time points (Time 2 and Time 3) in individual participants in the treatment group. However, as can be seen in Figure 2, the level of problematic assumptions, death anxiety, and psychological distress in cancer patients and their family caregivers assigned to the control group remained considerably high over time.

## 4. Discussion

The purpose of the current study was to examine the effects of REHCT on problematic assumptions, death anxiety, and psychological distress in a sample of cancer patients and their family caregivers in Nigeria. No baseline differences in problematic assumptions, death anxiety, and psychological distress were observed in cancer patients or their family caregivers in the treatment and control groups. The findings confirm earlier observations and reports that patients suffering from terminal illnesses and their family caregivers have unyielding self-talk, worries, fears, suspicions, psychological pain, and distress [19,24,25,44,45,46].

The study found that an REHCT intervention program had significant effects on problematic assumptions, death anxiety, and psychological distress among the cancer patients and their family caregivers compared with the control group both at the end of the intervention and at follow-up meetings. These findings support previous research that demonstrates the efficacy of a REBT approach and other cognitive behavior therapy techniques in helping patients and caregivers to manage health-related symptoms and behaviors [24,25,28,29,30,31,35,36,47,48,49,50].

The data from this study also support previous reports that recommend the use of evidence-based cognitive behavioral techniques in hospice care for individuals with life-threatening illnesses [51]. The findings from the current study also confirm the argument by Ellis and Bernard that the reputation and clinical utility of REBT theory in various cultures, and its increasing application to contemporary living, show that it continues to be vital and dynamic [23].

The preliminary evidence from this study suggests that large-scale REHCT interventions are urgently needed to address the psychological problems of cancer patients in Nigeria. As more cancer patients approach the end of their lives, cognitive behavioral intervention based on the principles and practice of REBT could become one of the most viable options to help these patients live with dying, and help their loved ones cope with the situation.

### 4.1. Implications for Practice

In view of the above findings, behavioral oncologists implementing REHCT must be aware that the meaning and type of control imposed by the patient could provide the blueprint for the relative efficacy of a specific intervention program—cognitive or behavioral, or even a combination of both. In addition, there is an array of negative emotions that can exacerbate for a terminal cancer patient the difficulties of dying an ‘appropriate death’. These emotions also limit their caregiver’s capability to develop coping skills for their own personal life. However, the REBT approach to hospice care helps the therapist specify the maladaptive cognitions of the dying patients and their caregivers that educe and maintain maladaptive emotions and behaviors, and provides a therapeutic model for cognitive, affective, and behavioral change.

Although it is important to show that an REHCT intervention can affect and change irrational assumptions, an REBT hospice care therapist also needs to show that the therapy influences the emotions and disturbances that the irrational assumptions are thought to cause. In this way, therapists can show that the intervention changed the problem and the irrational assumptions that led to the problem. In fact, the researchers feel that both types of measures are very much needed in the design of future studies in REHCT interventions. The researchers hope that future studies will address any limitations arising from the measures used in the current study given the many facets of irrational assumptions, emotional distress, psychopathology, or behavior of patients with various life-threatening illnesses. Certain sociocultural variables are also likely to influence the patterns of a patient’s problematic assumptions, emotional distress, psychopathology, or behavior about death and dying, or life and living. For this reason, REBT researchers must take into account the different sociocultural variables that could bias a client’s full disclosure of their concerns in an REHCT intervention program.

This study provides baseline data for future researchers and also provides a new direction in end-of-life care, patient education, and counseling in Nigeria. The researchers endeavored to make these findings as robust as possible by using item statements derived from irrational assumptions typical of patients with terminal illness, by implementing expert-consensus validation procedures, and by systematically controlling study error rates. The researchers were also alert to the degree with which the statements in the measures were in line with the purpose of the current study, and the degree with which the items on participant’s assumptions were also problematic, and considered the patterns of distress of the cancer patients and their family members within the Nigerian context during trial testing of the measures.

The researchers would like to suggest that future studies should make use of measures of disturbance and psychopathology, for instance, translated into other native languages while working with terminal cancer patients in different parts of the world. Overall, the current study with its preliminary evidence-based literature should serve as an invaluable resource for end-of-life education and counseling of cancer patients and their family caregivers in Nigeria.

### 4.2. Limitations of the Study

Despite the study’s positive outcomes, there are some limitations. An important limitation is the lack of data for other characteristics of the participants which might affect the result—e.g., educational status, spirituality, financial status, etc.

In addition, some might argue that the sample size in this study is small. However, there is evidence [43] that a REBT intervention is not affected by small sample size, but what is important is the therapist’s expertise in the therapeutic relationship.

## 5. Conclusions

The inability to accept the inevitability of death by a terminally ill person and their family caregivers is a psychological burden for both the patient and their caregivers. The person with cancer and their caregivers may show common reactions such as problematic assumptions, psychological distress, and anxiety. Empirical evidence from the current study strengthens the belief that REBT designed for hospice care intervention is effective in reducing these psychological impacts by facilitating a creative coping strategy for the cancer patients and their family caregivers. The study, therefore, highlights the need for follow-up studies both in Nigeria and in other parts of the world by REBT practitioners with an adequate background in behavioral oncology. These would assess the different psychosocial concerns of patients with life-threatening illness and their families, and attempt to use REHCT in resolving such problems.

There are several REBT techniques that can be used in hospice care interventions by oncology nurses and other behavioral oncologists in that REHCT is a humanistic-existential, cognitive behavioral, and family-centered approach to hospice care intervention. The researchers therefore encourage future researchers, oncology nurses, and other behavioral oncologists to test empirically the effectiveness of REHCT because the evidence suggests that it is effective in helping cancer patients and their caregivers cope with their situation.

The researchers hope that their findings will influence future interventions in terminal care for a different patient population in multicultural societies such as Nigeria. However, future studies should adhere to CONSORT guidelines and include the following: a clear indication of the benchmark values that were used as inclusion criteria, and how many sessions were delivered to participants in the treatment group; the timing of the assessments and how these times were chosen; the consistency values of the measures used and the sample sizes of patients and family members; details of how places were selected for advertising for participants; an explanation of when and how screening was performed for the participants; any limitations on recruitment; and an explanation of how the intervention manual was made, in which language(s), with which process and with the involvement of what kind of professionals; and whether it was pilot tested, and a more detailed explanation of the control intervention.

## Figures and Tables

**Table 1:** Sociodemographic information of the participants.

Characteristics		Cancer Patients (CP) *N* (%)	Treatment Group (CP) *N* (%)	Control Group (CP) *N* (%)	Primary Caregivers (PC) *N* (%)	Treatment Group (PC) *N* (%)	Control Group (PC) *N* (%)
Age of Participants		48.33 ± 6.55 ^a^	48.33 ± 6.17 ^a^	48.32 ± 6.43 ^a^	55.75 ± 3.01 ^a^	55.75 ± 3.03 ^a^	55.74 ± 3.05 ^a^
Gender	Male	4 (12.50)	2 (12.50)	2 (12.50)	8 (15.38)	4 (15.38)	4 (15.38)
	Female	28 (87.50)	14 (87.50)	14 (87.50)	44 (84.62)	22 (84.62)	22 (84.62)
State of Resident	Enugu	8 (25.00)	4 (12.50)	4 (12.50)	12 (23.08)	6 (23.08)	6 (23.08)
	Imo	12 (37.50)	7 (43.75)	5 (31.25)	20 (38.46)	11 (42.30)	9 (34.62)
	Anambra	12 (37.50)	5 (31.25)	7 (43.75)	20 (38.46)	9 (34.62)	11 (42.30)
Setting	Rural	20 (62.50)	11 (68.75)	9 (56.25)	32 (61.54)	20 (76.92)	12 (46.15)
	Urban	12 (37.50)	5 (31.25)	7 (43.75)	20 (38.46)	6 (23.08)	14 (53.85)
Type of Cancer	Breast	20 (62.50)	10 (62.50)	10 (62.50)	-	-	-
	Cervical	8 (25.00)	3 (18.75)	5 (31.25)	-	-	-
	Prostate	4 (12.50)	3 (18.75)	1 (6.25)	-	-	-

^a^ Mean age (SD) of participants, SD = Standard Deviation, CP = Cancer Patients, PC = Primary Caregivers.

**Table 2:** Summary statistics from repeated measures analysis of variance (ANOVA) on cancer patients’ level of problematic assumptions, death anxiety, and psychological distress by treatment condition and time.

Outcomes	Treatment Group, *N* = 16			Control Group, *N* = 16			*Df*	*F*	*Sig.*	$\eta_{p}^{2}$
	Time 1	Time 2	Time 3	Time 1	Time 2	Time 3				
	M (SD)	M (SD)	M (SD)	M (SD)	M (SD)	M (SD)				
**CPFCAQ**	73.69 ± 2.68	-	-	73.25 ± 3.32	-	-	(1, 30)	0.17	0.68	0.01
	-	26.94 ± 5.45	-	-	73.69 ± 1.89		(1, 30)	1081.90	0.00	0.98
	-	-	18.88 ± 1.15	-	-	74.45 ± 1.26	(1, 30)	15,919.26	0.00	0.99
**DAQ**	71.00 ± 7.75	-	-	70.69 ± 7.67		-	(1, 30)	0.01	0.91	0.00
	-	28.81 ± 5.75	-	-	73.69 ± 2.68	-	(1, 30)	851.80	0.00	0.97
	-	-	18.00 ± 1.79	-	-	74.56 ± 1.03	(1, 30)	11,213.52	0.00	0.99
***K*_10_**	49.94 ± 0.25		-	49.00 ± 2.71	-	-	(1, 30)	1.90	0.18	0.06
	-	16.00 ± 4.21	-	-	49.94 ± 0.25	-	(1, 30)	1094.14	0.00	0.98
	-	-	14.38 ± 2.55	-	-	49.44 ± 0.89	(1, 30)	2676.16	0.00	0.99

SD = Standard Deviation, *Df* = Degrees of Freedom, *Sig.* = Level of Significance, CPFCAQ = Cancer Patients’ and Family Caregivers’ Assumptions Questionnaire, DAQ = Death Anxiety Questionnaire, *K*_10_ = Kessler Psychological Distress Scale, $\eta_{p}^{2}$ = Partial Eta Squared.

**Table 3:** Summary statistics from repeated measures ANOVA on effects of a rational emotive hospice care therapy (REHCT) intervention on family caregivers’ problematic assumptions, death anxiety, and psychological distress by treatment condition and time.

Outcomes	Treatment Group, *N* = 26			Control Group, *N* = 26			*Df*	*F*	*Sig.*	$\eta_{p}^{2}$
	Time 1	Time 2	Time 3	Time 1	Time 2	Time 3				
	M (SD)	M (SD)	M (SD)	M (SD)	M (SD)	M (SD)				
**CPFCAQ**	74.73 ± 0.72	-	-	74.69 ± 0.79	-	-	(1, 50)	0.03	0.86	0.00
	-	31.58 ± 7.32	-	-	74.85 ± 0.37	-	(1, 50)	831.45	0.00	0.95
	-	-	18.96 ± 1.22	-	-	74.46 ± 1.36	(1, 50)	28,962.88	0.00	0.99
**DAQ**	74.81 ± 0.49	-	-	74.35 ± 1.44	-	-	(1, 50)	2.93	0.13	0.05
	-	33.50 ± 7.51	-	-	74.00 ± 3.06	-	(1, 50)	608.83	0.00	0.93
	-	-	18.12 ± 1.88	-	-	74.65 ± 1.06	(1, 50)	16,194.57	0.00	0.99
***K*_10_**	49.92 ± 0.27	-	-	49.88 ± 0.33	-	-	(1, 50)	0.21	0.65	0.00
	-	15.04 ± 4.99	-	-	49.50 ± 1.96	-	(1, 50)	963.96	0.00	0.95
	-	-	13.50 ± 2.82	-	-	49.81 ± 0.63	(1, 50)	3741.25	0.00	0.99

## References

[1] Lightfoot N., Steggles S., Gauthier–Frohlick D., Arbour-Gagnon R., Conlon M., Innes C., O’Bonsawin L., Merali H. (2005). Psychological, physical, social, and economic impact of travelling great distances for cancer treatment. Curr. Oncol..

[2] Girgis A., Lambert S., Johnson C., Waller A., Currow D. (2013). Physical, Psychosocial, Relationship, and Economic Burden of Caring for People with Cancer: A Review. J. Oncol. Pract..

[3] Brown M.L., Lipscomb J., Snyder C. (2001). The burden of illness of cancer: Economic cost and quality of life. Annu. Rev. Public Health.

[4] Girgis A., Lambert S.D. (2009). Caregivers of cancer survivors: The state of the field. Cancer Forum.

[5] Mitschke D.B. (2008). Cancer in the family: Review of the psychosocial perspectives of patients and family members. J. Fam. Soc. Work.

[6] Hagedoorn M., Sanderman R., Bolks H.N., Tuinstra J., Coyne J.C. (2008). Distress in couples coping with cancer: A meta-analysis and critical review of role and gender effects. Psychol. Bull..

[7] Resendes L.A., McCorkle R. (2006). Spousal responses to prostate cancer: An integrative review. Cancer Investig..

[8] Janda M., Steginga S., Dunn J., Langbecker D., Walker D., Eakin E. (2008). Unmet supportive care needs and interest in services among patients with a brain tumour and their carers. Patient Educ. Couns..

[9] Nijboer C., Triemstra M., Tempelaar R., Sanderman R., van den Bos G.A. (1999). Determinants of caregiving experiences and mental health of partners of cancer patients. Cancer.

[10] Couper J.W., Bloch S., Love A., Duchesne G., Macvean M., Kissane D.W. (2006). The psychosocial impact of prostate cancer on patients and their partners. Med. J. Aust..

[11] Northouse L.L., Mood D., Templin T., Mellon S., George T. (2000). Couples’ patterns of adjustment to colon cancer. Soc. Sci. Med..

[12] Szucs T.D. (2000). Medical economic considerations of supportive cancer care. Int. J. Antimicrobia. Agents.

[13] Payne S., Jarrett N., Jeffs D. (2000). The impact of travel on cancer patients’ experiences of treatment: A literature review. Eur. J. Cancer Care.

[14] Baider L., Kaufman B., Ever–Hadani P., De-Nour A.K. (1996). Coping with additional stresses: Comparative study of healthy and cancer patient new immigrants. Soc. Sci. Med..

[15] Schumacher K.L., Stewart B.J., Archbold P.G., Dodd M.J., Dibble S.L. (2000). Family caregiving skill: Development of the concept. Res. Nurs. Health.

[16] Abiodun O.A., Olu-Abiodun O.O., Sotunsa J.O., Oluwole F.A. (2014). Impact of health education intervention on knowledge and perception of cervical cancer and cervical screening uptake among adult women in rural communities in Nigeria. BMC Public Health.

[17] Rotimi O., Abdulkareem F.B. (2014). Fifty-three years of reporting colorectal cancer in Nigerians—A systematic review of the published literature. Niger. Postgrad. Med. J..

[18] Jedy-Agba E., Curado M.P., Ogunbiyi O., Oga E., Fabowale T., Igbinoba F., Osubor G., Otu T., Kumai H., Koechlin A. (2012). Cancer incidence in Nigeria: A report from population-based cancer registries. Cancer Epidemiol..

[19] Doka K.J. (2014). Counseling Individuals with Life-Threatening Illness.

[20] Ellis A. (1957). Rational Psychotherapy and Individual Psychology. J. Individ. Psychol..

[21] Ellis A. (1958). Rational psychotherapy. J. Gen. Psychol..

[22] Ellis A. (1962). Reason and Emotion in Psychotherapy.

[23] Ellis A., Bernard M.E. (1985). What is Rational-Emotive Therapy (RET)?. Clinical Applications of Rational-Emotive Therapy.

[24] Sobel H.J., Bernard M.E., Ellis A. (1985). Rational living with dying. Clinical Applications of Rational-Emotive Therapy.

[25] Morris S. (2008). Overcoming Grief: A Self-Help Guide Using Cognitive Behavioral Techniques.

[26] Kosminsky P. (2016). CBT for grief: Clearing cognitive obstacles to healing from loss. J. Rational Emot. Cogn. Behav. Ther..

[27] Morris S. The Psychology of Grief: Applying Cognitive and Behavior Therapy Principles. https://www.psychology.org.au/Content.aspx?ID=4088.

[28] Kavanagh D.J. (1990). Towards a cognitive-behavioural intervention for adult grief reactions. Br. J. Psychiatry.

[29] Malkinson R. (2001). Cognitive-behavioral therapy of grief: A review and application. Res. Soc. Work Pract..

[30] Boelen P.A., de Keijser J., van den Hout M.A., van den Bout J. (2007). Treatment of complicated grief: A comparison between cognitive-behavioral therapy and supportive counseling. J. Consult. Clin. Psychol..

[31] McLeod S. Cognitive-Behavioral Therapy. http://www.simplypsychology.org/cognitive-therapy.html.

[32] Jackson E. (1977). Counseling the dying. Death Educ..

[33] LeShan L., LeShan E., Pearson L. (1969). Psychotherapy and the dying patient. Death and Dying: Current Issues in the Treatment of the Dying Person.

[34] LeShan L., LeShan E. (1961). Psychotherapy and the patient with a limited life span. Psychiatry.

[35] Sobel H.J. (1981). Behaviour Therapy in Terminal Care. Behaviour Therapy in Terminal Care: A Humanistic Approach.

[36] Janis I.L., Mann L. (1977). Decision Making: A Psychological Analysis of Conflict, Choice, and Commitment.

[37] Herz B.F., Carter B., McGoldrick M. (1989). The impact of death and serious illness on the family life cycle. the Changing Family Life Cycle: Framework for Family Therapy.

[38] Conte H.R., Weiner M.B., Plutchik R. Measuring Death Anxiety. http://www.blinn.edu/socialscience/LDThomas/Feldman/Handouts/1904hand.htm.

[39] Kessler R.C., Andrews G., Colpe L.J., Hiripi E., Mroczek D.K., Normand S.L., Walters E.E., Zaslavsky A.M. (2002). Short screening scales to monitor population prevalences and trends in non-specific psychological distress. Psychol. Med..

[40] David D., Kangas M., Schnur J.B., Montgomery G.H. (2004). REBT Depression Manual/Protocol: Managing Depression Using Rational Emotive Behavior Therapy.

[41] Ellis A., Grieger R. (1977). Handbook of Rational-Emotive Therapy.

[42] Walen S.R., Di Giuseppe R., Dryden W. (1992). A Practitioner’s Guide to Rational Emotive Therapy.

[43] Eseadi C., Anyanwu J.I., Ogbuabor S.E., Ikechukwu-Ilomuanya A.B. (2016). Effects of cognitive restructuring intervention program of rational-emotive behavior therapy on adverse childhood stress in Nigeria. J. Rational Emot. Cogn. Behav. Ther..

[44] Mayo Foundation for Medical Education and Research Terminal Illness: Supporting A Terminally Ill Loved One. http://www.mayoclinic.org/healthy-lifestyle/end-of-life/in-depth/grief/art-20047491.

[45] National Cancer Institute End-of-Life Care for People Who Have Cancer. http://www.cancer.gov/about-cancer/advanced-cancer/care-choices/care-fact-sheet.

[46] WebMD Hospice Care: Topic Overview. http://www.webmd.com/balance/tc/hospice-care-topic-overview.

[47] Ellis A., Sobel H.J. (1981). The rational-emotive approach to thanatology. Behavior Therapy in Terminal Care.

[48] Sobel H.J., Worden J.W. (1982). Helping Cancer Patients Cope: A Problem-Solving Intervention for Health-Care Professionals.

[49] Weisman A.D. (1979). Coping with Cancer.

[50] Weisman A.D. (1980). What do elderly, dying patients want, anyway?. J. Geriatr. Psychiatry.

[51] American Cancer Society Hospice Care. http://www.cancer.org/acs/groups/cid/documents/webcontent/002868-pdf.pdf.

